# The dose-dependent effect of 1.5-GHz microwave exposure on spatial memory and the NMDAR pathway in Wistar rats

**DOI:** 10.1007/s11356-022-24850-4

**Published:** 2022-12-27

**Authors:** Hui Wang, Lequan Song, Li Zhao, Haoyu Wang, Xinping Xu, Ji Dong, Jing Zhang, Binwei Yao, Xuelong Zhao, Ruiyun Peng

**Affiliations:** Beijing Institute of Radiation Medicine, 27 Taiping Road, Beijing, People’s Republic of China

**Keywords:** Microwave, Dose-dependent effect, Spatial memory, Histopathology, NMDAR, Hippocampus, Cognitive functions

## Abstract

A certain power of microwave radiation could cause changes in the nervous, cardiovascular, and other systems of the body, and the brain was a sensitive target organ of microwave radiation injury. Studies have shown that microwaves can impair cognitive functions in humans and animals, such as learning and memory, attention, and orientation. The dose-dependent effect of microwave radiation is still unclear. Our study aimed to investigate the effects of 1.5-GHz microwaves with different average power densities on locative learning and memory abilities, hippocampal structure, and related N-methyl D-aspartate receptor (NMDAR) signalling pathway proteins in rats. A total number of 140 male Wistar rats were randomly divided into four groups: S group (sham exposure), L5 group (1.5-GHz microwaves with average power density = 5 mW/cm^2^), L30 group (1.5-GHz microwaves with average power density = 30 mW/cm^2^), and L50 group (1.5-GHz microwaves with average power density = 50 mW/cm^2^). Changes in spatial learning and memory, EEG activity, hippocampal structure, and NMDAR signalling pathway molecules were detected from 6 h to 28 d after microwave exposure. After exposure to 1.5-GHz microwaves, rats in the L30 and L50 groups showed impaired spatial memory, inhibited EEG activity, pyknosis and hyperchromatism of neuron nucleus, and changes in NMDAR subunits and downstream signalling molecules. In conclusion, 1.5-GHz microwaves with an average power density of 5, 30, and 50 mW/cm^2^ could induce spatial memory dysfunction, hippocampal structure changes, and changes in protein levels in rats, and there was a defined dose-dependent effect.

**Supplementary Information:**

The online version contains supplementary material available at 10.1007/s11356-022-24850-4.

## Introduction

With the increasing use of electromagnetic equipment in the fields of communications, agriculture, industry, and military affairs, the impact of electromagnetic radiation on human health has received widespread attention(Leung et al. 2011; Cabré-Riera et al. 2021). Studies have shown that microwaves can affect the brain, heart, and reproductive system, and the brain is the most sensitive organ (Eliyahu et al. 2006; Narayanan et al. 2010; Fragopoulou et al. 2018). Electromagnetic waves are considered a fourth pollutant by the World Health Organization (WHO) and classified as “probably carcinogenic to humans” (Group 2B) by the International Agency for Research on Cancer (IARC) (Baan et al. 2011). L-band microwaves are defined as electromagnetic radiation with a frequency of 1–2 GHz (Wigneron et al. 2007). Compared to the S- and X-bands, L-band microwaves have a lower frequency, longer wavelength and deeper penetration. Studies have shown that 1.5-GHz microwaves can impair learning and memory function and damage the hippocampal structure in rats (Tan et al. 2021; Zhu et al. 2021). The microwaves emitted from mobile phones, wireless hotspots, and mobile communication base stations are usually in a frequency range of 800 MHz–2.6 GHz, which places them mainly within the L-band. Epidemiological studies have found that microwave radiation can cause various neurological symptoms, such as dizziness, headache, insomnia, and memory loss(Hutter et al. 2006; Schmid et al. 2012). However, research on the dose-dependent effect of L-band microwave radiation damage is still lacking. Therefore, our study used rats irradiated with 1.5-GHz microwaves at different power densities to characterize the underlying dose-dependent effects and mechanism. In addition, N-methyl D-aspartate receptor (NMDAR) signal transduction proteins, such as GluN1, GluN2A, GluN2B, PSD-95, CaMKII, and CREB, were measured to identify molecules that may be sensitive to 1.5-GHz microwave exposure.

## Materials and methods

### Ethical approval

The animal work in this study was approved by the Animal Care and Use Committee of the Academy of Military Medical Science (IACUC-AMMS-2020–780). It was carried out on the basis of the National Institute of Health Guide for the Care and Use of Laboratory Animals (NIH Publication No. 85–23, revised 1996). Our study was conducted in compliance with the Animal Research: Reporting In Vivo Experiments (ARRIVE) guidelines (http://www.nc3rs.org.uk/page.asp?id=1357).

### Animal grouping

A total number of 140 male Wistar rats (200 ± 20 g, 6–8 weeks) were provided by Beijing Vital River Laboratory Animal Technology Co., Ltd. They were raised in specific-pathogen-free (SPF)-level animal facilities and maintained at a constant temperature of 22–24 °C, 40–60% humidity, and a 12/12-h light/dark cycle (lights on from 7 a.m. to 7 p.m.).

The 1.5-GHz microwave belongs to the L-band. The power densities of the microwaves used in the study were 5 mW/cm^2^, 30 mW/cm^2^, and 50 mW/cm^2^. The letter “L” represents the 1.5-GHz exposure group, and the numbers “5,” “30,” and “50” represent the power density of each group.

All rats were randomly divided into four groups: (1) the sham radiation group (the S group), (2) rats exposed to 5 mW/cm^2^ microwave radiation with a frequency of 1.5-GHz (the L5 group), (3) rats exposed to 30 mW/cm^2^ microwave radiation with a frequency of 1.5-GHz (the L30 group), and (4) rats exposed to 50 mW/cm^2^ microwave radiation with a frequency of 1.5-GHz (the L50 group).

### Experimental design

The microwave exposure system that we used in this study has been described in detail in our previous article(Wang et al. 2013a). In short, the microwave energy was transmitted into an electromagnetically shielded chamber through a rectangular waveguide and the 16 dB standard gain horn antenna. The rats of the S, L5, L30, and L50 groups were exposed to 1.5-GHz microwaves with average power densities of 0 mW/cm^2^, 5 mW/cm^2^, 30 mW/cm^2^, and 50 mW/cm^2^, respectively, for 6 min. The rats were placed in a container made of plexiglass. During microwave exposure, the rat container was rotated at a constant speed to ensure that each rat received the same radiation dose. The average power densities were measured by a GX12M1CHP power meter (Guanghua Microelectronics Instruments, Hefei, China) and GX12M30A power heads.

The specific absorption rate (SAR) was calculated according to the method described in our previous paper(Tan et al. 2017). Through the simulation platform Empire: IMST-Empire v-4.10 (GmbH, Kamp-Lintfort, Germany), the finite-difference time-domain method was used to calculate the special absorption rate of each rat under plane wave exposure, which was sliced by magnetic resonance imaging (MRI). The established rat model for calculating the SAR is based on 370 g male Sprague–Dawley rats with 36 different tissues. The original resolution of the model is 0.39 × 0.39 × 1 mm, which yields a three-dimensional array containing 6.6 million pixels. These cubes are labelled to identify the type of organization. Under this condition, we normalized the model to 230 g, which was the average mass of the rats used in the experiment; the voxel size was 0.30 × 0.30 × 0.80 mm. On this basis, we calculated the average SAR of the brain; we found that the average brain SARs of the S, L5, L30, and L50 groups were 0, 1.85, 11.1, and 18.5 W/kg, respectively. Due to the uncertainty of the field value; amplifier drift and changes in position, direction, attitude and anatomical structure, there was uncertainty in the SAR; however, this uncertainty was less than 2 dB.

### Temperature measurement

Before and after microwave radiation, the rectal temperature (*n* = 5) of the rats in each group was measured with a portable intelligent digital thermometer (TH212, China), and the body surface temperature (*n* = 5) of the rats in each group was measured with an infrared thermal imager (FLIR, USA).

### Spatial learning and memory test

The Morris water maze (MWM, Beijing Sunny Instrument, China) was used to detect the spatial learning and memory ability of rats; the experimental procedure has been described in the literature (Qiao et al. 2014, Vorhees and Williams 2014). The MWM was divided into four quadrants, and the escape platform was placed in the middle of the first quadrant, 1–2 cm below the water surface. The water in the MWM was untreated tap water. The pool walls and the escape platform were black; therefore, in the dark environment, the rats could not see the platform. The high contrast between the environment and the rats’ body color enabled the camera to capture their trajectory more clearly. Before microwave exposure, MWM training was performed for three consecutive days. The rats were placed in the water, facing the nearest wall of the pool, at four points in the non-platform quadrants in a certain order each day; in this manner, the rats were familiarized with the task of searching for the platform. If a rat found the platform within 60 s, we stopped the timer and let the animal stay on the platform for 15 s. If the rat did not find the platform within 60 s, we recorded an escape latency of 60 s and guided the rat to the platform, where it was then allowed to stay for 15 s. The spatial reference memory test was conducted at 6 h, 1 d, 2 d, 3 d, 7 d, and 14 d after microwave radiation. The latency of each rat to find the platform was recorded in four trials per day, and the average escape latency (AEL) in the MWM was calculated for each rat. At 4 d after microwave radiation, the spatial probe test was carried out; for this test, the platform was removed, and the number of times the rats crossed the previous platform location in one minute was recorded. The time spent in the platform quadrant during the spatial reference memory test and the spatial probe test was also recorded. The spatial reference memory test and the spatial probe test were used to assess the spatial memory ability of the rats in each group.

### Electroencephalograph (EEG) recording and analysis

At 6 h, 7 d, 14 d, and 28 d after microwave irradiation, rat EEGs were collected and quantitatively analysed using a multi-conductor physiological recorder (Biopac Company, USA). Five rats were selected from each group and injected with 1% pentobarbital sodium 0.5 ml/100 g IP. The EEG signals were recorded using needle electrodes made of stainless steel. The electrode needle was placed under the scalp 1 mm next to the midpoint of the connecting line (equivalent to 1 mm next to the posterior sagittal suture of the coronal suture). A reference electrode was placed at the edge of the ear (Weiergräber et al. 2016). The EEG signals of the rats were recorded for 3 min. We recorded EEGs from the same animals at different time points after microwave exposure. The animals did not undergo surgery. The electrodes were connected to the EEG amplifier to filter the collected signals into signals of different bands. The sampling frequency was 100 Hz. The MP-150 multi-conductor physiological recording and analysis system was used to process the EEG signals of the rats and analyse the changes in the power of the 4 types of brain waves: α (12–30 Hz), β (8–12 Hz), θ (4–8 Hz), and δ (1–4 Hz). The electrodes were connected to an EEG amplifier to filter the collected signals into different bands. The sampling frequency was 100 Hz.

### Histopathological examination

At 6 h, 7 d, 14 d, and 28 d after exposure, five rats in each group were randomly selected and anaesthetized with sodium pentobarbital (50 mg/kg, IP). The rats were sacrificed with 1% sodium pentobarbital solution for pathological examination. The brain of each rat was removed, and the left half of the brain was fixed in 10% neutral buffered formalin. The right half was stored in the refrigerator at − 80 °C for subsequent experiments. From the left half of each brain, a tissue sample containing the hippocampus was removed and made into paraffin-embedded sections (Qin et al. 2018). The prepared paraffin-embedded sections were dewaxed with gradient ethanol and xylene in water and then immersed in haematoxylin for 5 min. The sections were decolorized with 1% hydrochloric acid in ethanol for 7 s and then re-dyed with eosin for 2 min. After dehydration with gradient ethanol and xylene, the slices were sealed with neutral gum and dried naturally. The hippocampal formation was observed under an optical microscope (Leica, Germany). Referring to the method in a previous paper(Tosta et al. 2019), we counted the deeply stained neuron nuclei in the hippocampus of each group of rats for relative quantitative analysis to evaluate the degree of hippocampal injury.

### Ultrastructure of hippocampal tissue

At 7 d after microwave exposure, ultrastructural injury to the hippocampus in each group was observed. The hippocampal tissue of the left hemisphere of rats was fixed overnight in 2.5% glutaraldehyde phosphate buffer, washed with 0.1 mol/l phosphate buffer, fixed with 1% osmium tetroxide and washed with double-distilled water. The fixed tissue was dehydrated with gradient ethanol, soaked with acetone and embedding solution, and cut into semi-thin sections (Tizro et al. 2019). After H&E staining, the tissues were located by light microscopy, and ultrathin sections were made to observe the ultrastructure of the hippocampus under a transmission electron microscope (TEM, Hitachi, Japan). Finally, quantitative analysis was performed by ImageJ 1.8.021 software to measure the thicknesses of hippocampal neurons’ postsynaptic densities (PSDs).

### Western blotting

Western blotting was performed using the Simple Western Jess system (ProteinSimple, USA), a combination of capillary electrophoresis and immunodetection techniques, following the manufacturer’s protocols (Beekman et al. 2018). The left hippocampal samples were collected and were frozen at − 80℃ at 6 h and 7 d after microwave exposure. The hippocampus was homogenized and lysed in a mixed liquid containing radio-immunoprecipitation assay (RIPA) lysis buffer and 1% protease inhibitor after all the samples were collected. The whole process was carried out at a low temperature (0–4℃). A fully automated western blotting was carried out on an array of protein samples after the protein concentration was determined with a bicinchoninic acid (BCA) protein assay kit. Proteins were detected with the following primary antibodies: PSD-95 (ab18258, Abcam), CaMKII (ab134041, Abcam), CREB (ab32515, Abcam), (ab181602, Abcam), GluN1 (MAB363, Millipore Sigma), GluN2A (MAB5216, Millipore Sigma) and GluN2B (ab93610, Abcam). Chemiluminescent signals were captured by a charge-coupled device (CCD) camera, and the resulting images were analysed by Compass software (ProteinSimple, USA) and expressed as peak intensities. For quantification, the areas under the protein peaks were normalized to GAPDH (ab181602, Abcam) and target protein loading controls.

### Statistical analysis

SPSS 25 software was used to process the data of this article. All data passed a normality test and are expressed as the mean ± standard error ($\overline{X }$± SE). A paired *t*-test was used to analyze the changes in rectal temperature and body surface temperature before and after microwave radiation. The results of the MWM and EEG were analysed by two-way repeated-measures ANOVA. Other data were analyzed by one-way ANOVA. Differences at *P* < 0.05 were considered significant. Symbols were assigned to each effect based on the comparison and the level of significance, as follows: ^*^P < 0.05 or ^**^P < 0.01 (vs. S), ^#^*P* < 0.05 or ^##^*P* < 0.01 (vs. L5), ^☆^*P* < 0.05 or ^☆☆^*P* < 0.01 (vs. L30). All statistical graphs were drawn by GraphPad Prism 8.

## Results

### Body surface temperature and core temperature

The results showed that the changes in rectal temperature and body surface temperature after microwave radiation were less than 1 °C (Table 1) (*P* > 0.05). Therefore, under the experimental conditions, the influence of microwave radiation was mainly a non-thermal effect.

**Table 1:** Rectal and body surface temperature before and after microwave radiation exposure in the 4 studied groups

Groups	Rectal temperature before microwave exposure (°C)	Rectal temperature after microwave exposure(°C)	Body surface temperature before microwave exposure (°C)	Body surface temperature after microwave exposure (°C)
S	38.73 ± 0.34	38.50 ± 0.41	34.63 ± 0.94	34.75 ± 0.66
L5	39.08 ± 0.33	38.78 ± 0.53	33.75 ± 0.69	33.85 ± 0.75
L30	39.03 ± 0.22	39.08 ± 0.30	34.15 ± 0.93	34.48 ± 0.75
L50	38.90 ± 0.32	39.23 ± 0.29	34.03 ± 0.30	34.75 ± 0.66*

^*^No significant difference in temperature before and after microwave exposure

### The spatial learning and memory ability of rats

**Fig. 1:**
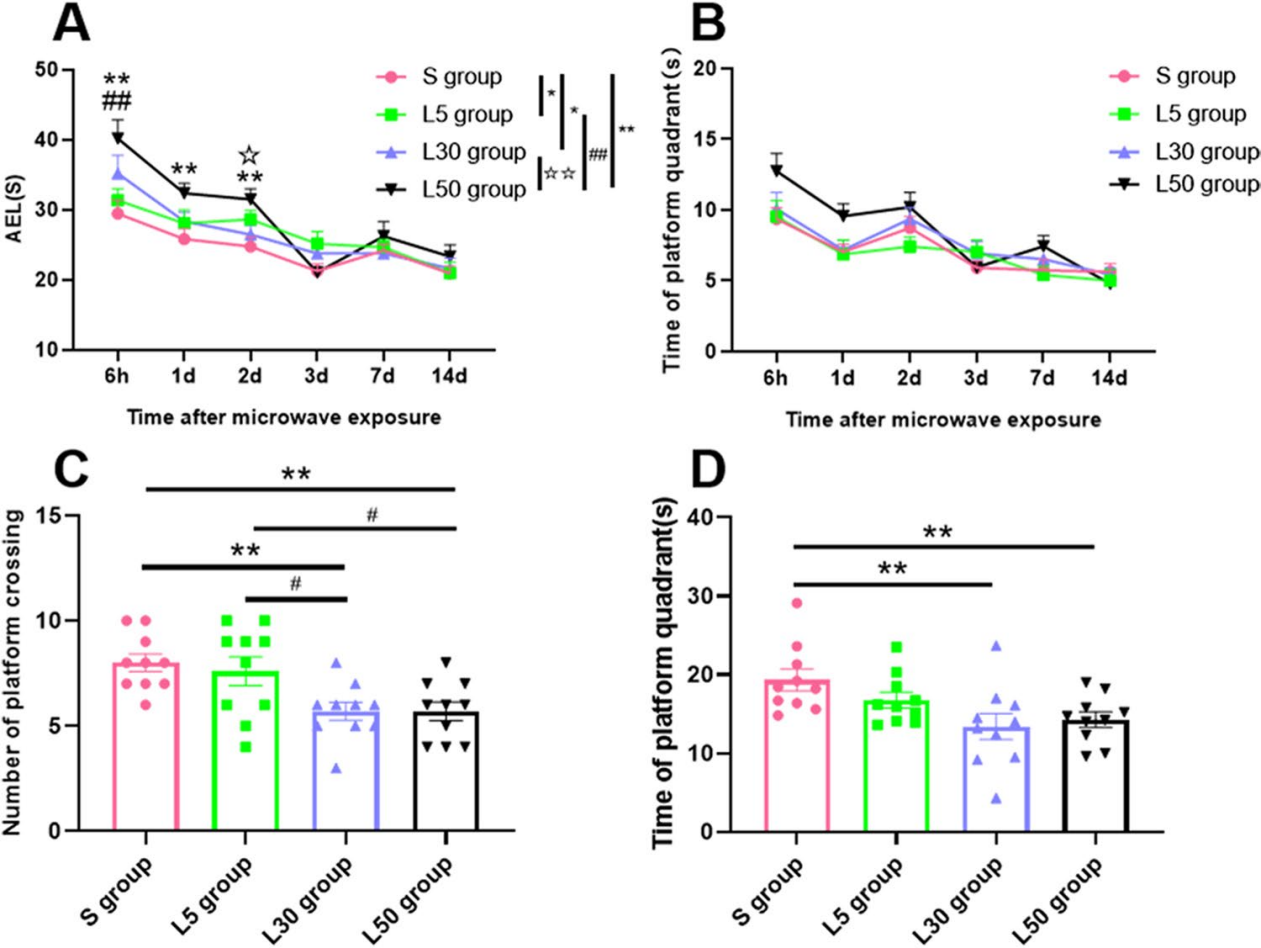
Analysis of the MWM test in rats. (**A**) Changes in the water maze AEL of rats 6 h–14 d after microwave exposure. (**B**) Changes in the time spent by rats in the platform quadrant of the water maze 6 h–14 d after microwave exposure. (**C**) The number of times that rats crossed the platform within 1 min in the spatial probe test. (**D**) The time spent in the platform quadrant within 1 min in the spatial probe test. Compared with the S group, * indicates *P* < 0.05, and ** indicates *P* < 0.01. Compared with the L5 group, ^#^ indicates *P* < 0.05. Compared with the L30 group, ^☆^ indicates *P* < 0.05. The colors of the points correspond to the groups. The overall group effects are indicated by the significance marks on the graphs

The results of the MWM navigation experiment (Fig. 1A) showed that the AEL of the L50 group was significantly longer than that of the S group at 6 h (*P* = 0.002, *n* = 10), 1 d (*P* = 0.005, *n* = 10) and 2 d (*P* = 0.001, *n* = 10) after 1.5-GHz microwave exposure. In addition, at 6 h after 1.5-GHz microwave exposure, the AEL of the L50 group was significantly longer than that of the L5 group (*P* = 0.008, *n* = 10). At 2 d after 1.5-GHz microwave exposure, the AEL of the L50 group was significantly longer than that of the L30 group (*P* = 0.014, *n* = 10). There were no significant changes in the AEL in any of the exposure groups at 3 d, 7 d or 14 d (*P* > 0.05, *n* = 10). Overall, the AELs of the S group (*P* = 0.000, *n* = 10), L5 group (*P* = 0.002, *n* = 10) and L30 group (*P* = 0.003, *n* = 10) were significantly lower than that of the L50 group. Compared with the S group, the AELs of the L5 group (*P* = 0.014, *n* = 10) and L30 group (*P* = 0.012, *n* = 10) increased significantly.

The time spent in the platform quadrant for each group was measured at 6 h–14 d after 1.5-GHz microwave exposure (Fig. 1B), and there was no significant difference among the groups at any time point (*P* > 0.05, *n* = 10). Additionally, there was no significant difference among the groups overall (*P* > 0.05, *n* = 10).

The results of the spatial probe test (Fig. 1C) showed that compared, with the S group, there were no significant changes in the numbers of rats crossing the platform in 1 min in the L5 group (*P* > 0.05, *n* = 10), whereas the number of platform crossings decreased significantly in the L30 group (*P* = 0.003, *n* = 10) and the L50 group (*P* = 0.003, *n* = 10). Additionally, compared with the L5 group, the number of platform crossings was significantly reduced in the L30 group (*P* = 0.012, *n* = 10) and L50 group (*P* = 0.012, *n* = 10). There were no significant differences in the number of platform crossings between the L30 group and the L50 group (*P* > 0.05, *n* = 10).

In the spatial probe test, the time spent in the platform quadrant was recorded (Fig. 1D). Compared with the S group, the L30 group (*P* = 0.002, *n* = 10) and L50 group (*P* = 0.009, *n* = 10) spent increased total amounts of time in the platform quadrant.

### EEG activity after 1.5-GHz microwave exposure

**Fig. 2:**
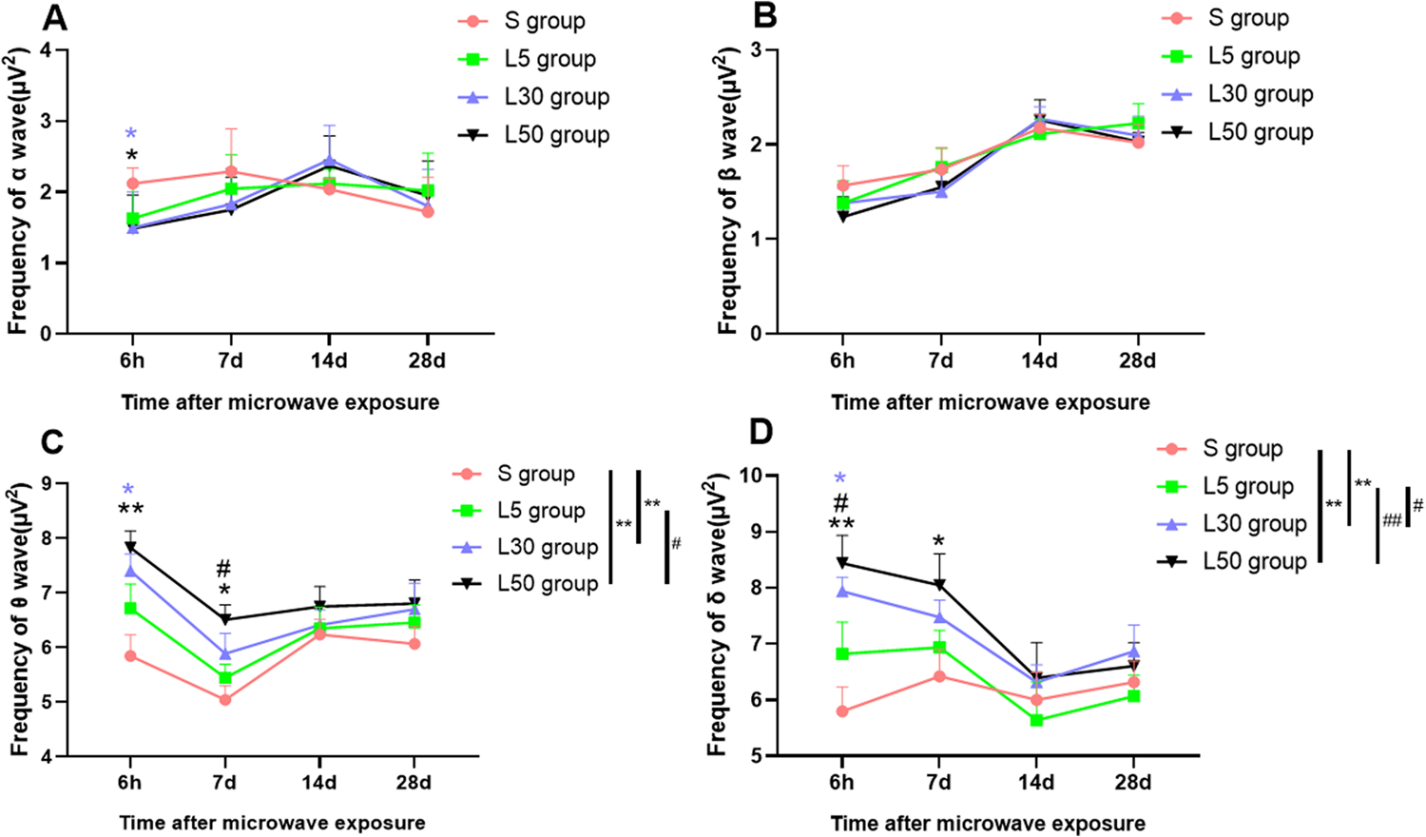
EEG power (μV^2^) in rats after 1.5-GHz microwave exposure. (**A**–**D**) Power variation of α, β, θ, and δ waves at different times after microwave exposure. Compared with the S group, * indicates *P* < 0.05, and ** indicates *P* < 0.01. Compared with the L5 group, ^#^ indicates *P* < 0.05. The colour of the mark is consistent with the grouping. The overall group effects are indicated by the significance marks on the graphs

The changes in α wave power in each group after exposure to 1.5-GHz microwaves are shown in Fig. 2A. At 6 h after exposure, compared with the S group, the α wave power of rats in the L30 and L50 groups was significantly reduced (*P* = 0.041, *n* = 5) (*P* = 0.036, *n* = 5).

The changes in β wave power after 1.5-GHz microwave exposure are shown in Fig. 2B; these changes were not significant (*P* > 0.05, *n* = 5).

The changes in θ wave power were follows (Fig. 2C). At 6 h after exposure, compared with the S group, the rats in the L30 group (*P* = 0.008, *n* = 5) and L50 group (*P* = 0.001, *n* = 5) showed significantly increased θ wave power. At 7 d after exposure, the θ wave power of the L50 group was significantly higher than that of the S group (*P* = 0.002, *n* = 5) and L5 group (*P* = 0.019, *n* = 5).

The changes in δ wave power were as follows (Fig. 2D). At 6 h after exposure, the δ wave power of the L50 group was significantly higher than that of the S group (*P* = 0.001, *n* = 5) or L5 group (*P* = 0.023, *n* = 5). Compared with the S group, the L30 group showed a significantly increase in the δ wave power (*P* = 0.004, *n* = 5). At 7 d after exposure, the L50 group had significantly higher δ wave power than the S group (*P* = 0.016, *n* = 5).

### Hippocampal structure of rats after 1.5-GHz microwave radiation

The normal hippocampal tissue structure of rats showed an orderly arrangement of neurons, light staining of nuclei and uniform eosinophilic cytoplasm. In the L5, L30, and L50 groups, different degrees of hippocampal injury occurred, all indicator for neuronal degeneration and necrosis mainly manifested as pyknosis and nuclear hyperchromacia, enhanced eosinophilic cytoplasm and granulosa cells in the dentate gyrus (DG) region, and neurons in the CA3 region suffered more severe injury.

**Fig. 3:**
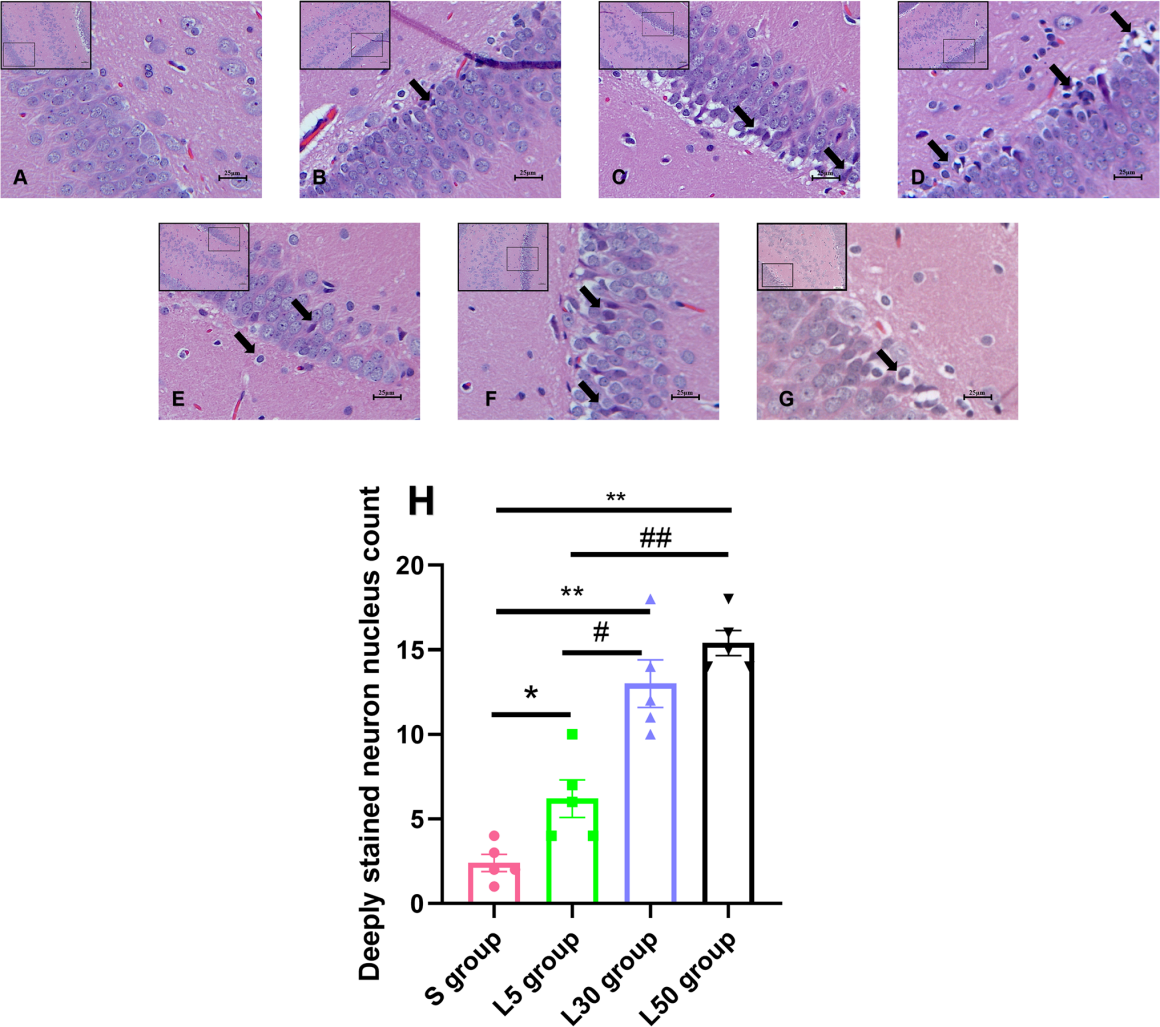
Pathological injuries in the hippocampal DG and CA3 regions of rats 6 h after L-band microwave exposure. (**A**) The hippocampal tissue structure was normal in the S group at 7 d after microwave exposure. (**B**–**D**) The hippocampal tissue structure of the L5 group, L30 group, and L50 group was injured to varying degrees at 7 d after microwave exposure. (**E**–**G**) Dynamic changes in hippocampal tissue structure at 6 h, 14 d, and 28 d after microwave exposure in the L50 group. (**H**) Quantitative analysis of deeply stained neuron nuclei. The boxes represent areas of structural impairment, and the arrows indicate deep staining of nuclear pyknosis and enhanced cytosolic eosinophilic neurons. Scale bars = 25 µm. Magnification power as: H&E.40

At 7 d after exposure, granulosa cells and neurons in the hippocampal DG area and CA3 area were normal in the S group (Fig. 3A). In the L5 group, there was occasional deep staining of nuclear pyknosis (Fig. 3B). In the L30 group, there were obvious lesions, with more pyknosis of neuron (Fig. 3C). The most serious injury was observed in the L50 group, which showed a large number of pyknotic neuronal nuclei and hyperchromatic, enhanced cytosolic eosinophils (Fig. 3D). Therefore, we further investigated the dynamic changes in hippocampal injury in the L50 group. At 6 h after microwave exposure, the hippocampal tissue structure was injured (Fig. 3E). At 7 d after exposure, the injury to the hippocampal tissue structure was at its most severe (Fig. 3D). At 14 d after exposure, the injury still existed but was partially recovered (Fig. 3F). At 28 d after exposure, the site of the injury had largely returned to normal, with only a few nuclei deeply stained (Fig. 3G).

Deeply stained neuron nuclei in the DG and CA3 regions were quantitatively analysed (Fig. 3H). Compared with the S group, the number of hyperchromatic neuronal nuclei increased in the L5 group (*P* = 0.017, *n* = 5), L30 group (*P* = 0.000, *n* = 5) and L50 (*P* = 0.000, *n* = 5) group. Compared with the L5 group, the number of hyperchromatic neuronal nuclei increased in the L30 group (*P* = 0.000, *n* = 5) and L50 group (*P* = 0.000, *n* = 5).

### Ultrastructural changes in the rat hippocampus after 1.5-GHz microwave exposure

**Fig. 4:**
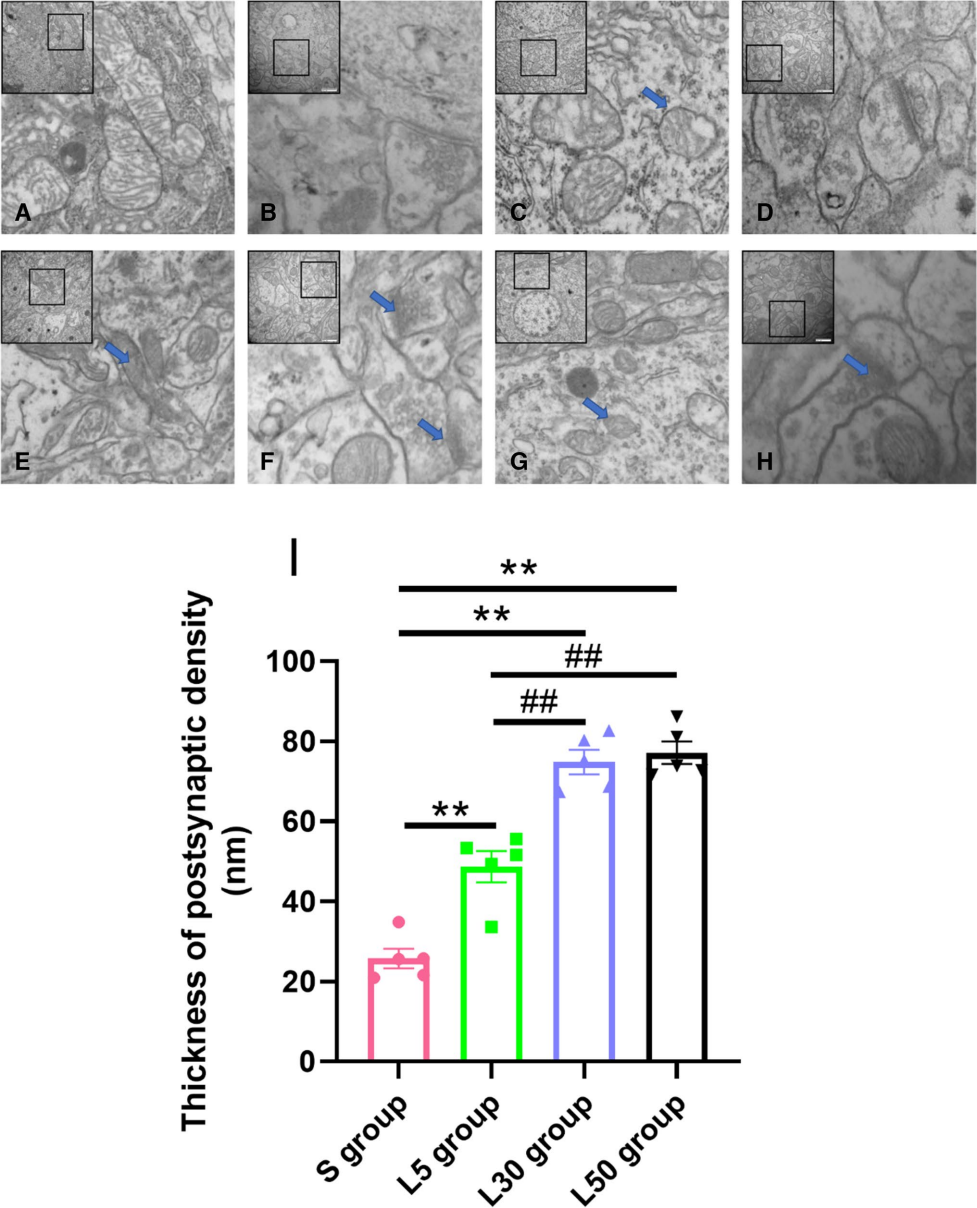
Ultrastructural changes in the hippocampus after 1.5-GHz microwave exposure. (**A**–**B**) The S group showed no changes in neurons or synapses. (**C**–**D**) The L5 group. (**E**–**F**) The L30 group. (**G**–**H**) The L50 group. In the radiation group, mitochondria were swollen, cristae were broken, and the PSD was thickened. As the dose increased, the injury became severe. The boxes represent areas of ultrastructural impairment, the blue arrows indicate swollen hollow mitochondria, and the black arrows indicate thickened synapses. (**I**) Quantitative analysis of PSD thickness. Compared with the S group, ** indicates *P* < 0.01. Compared with the L5 group, ^##^ indicates *P* < 0.01. Scale bars = 250 or 500 nm

The ultrastructure results are shown in Fig. 4. At 7 d after exposure, the ultrastructure of the hippocampus in the S group was normal, the number of vesicles did not increase, the synaptic space was clearly visible, and the postsynaptic density was not thickened. In addition, the mitochondria and nuclei in neurons were clearly visible, and there was no widening change in the nuclear membrane space. The size and shape of the mitochondria and rough endoplasmic reticulum in the cytoplasm showed no significant changes (Fig. 4A–B). In the L5, L30, and L50 groups, the injury was mainly characterized by the accumulation of presynaptic vesicles, blurred synaptic space and thickening of postsynaptic density. Mitochondria in neurons were swollen and even empty (Fig. 4C–H). The thickness of the PSD was quantitatively analyzed, and the results were as follows (Fig. 4I). Compared with the S group, the thickness of the PSD in the L5 group (*P* = 0.000, *n* = 5), L30 group (*P* = 0.000, *n* = 5) and L50 group (*P* = 0.000, *n* = 5) increased significantly. Compared with the L5 group, the thickness of the PSD of the L30 group (*P* = 0.000, *n* = 5) and L50 group (*P* = 0.000, *n* = 5) increased significantly. There were no significant differences between the L30 group and the L50 group (*P* > 0.05, *n* = 5).

In summary, compared with the S group, the ultrastructure of synapses and neurons was slightly damaged in the L5 group. However, more obvious injuries were observed in the L30 and L50 groups. The L30 group and the L50 group both showed more serious damage than the L5. However, there was no significant difference between the L30 group and the L50 group.

### NMDAR subunits and related signalling molecules change

**Fig. 5:**
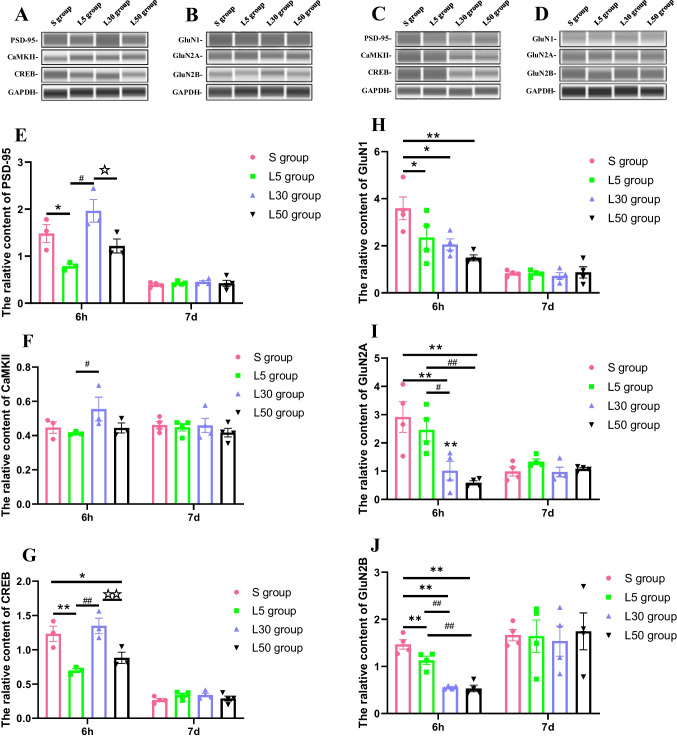
Expression of NMDAR subunits and downstream signalling pathway molecules after microwave exposure. (**A**–**B**) The protein expression levels of PSD-95, CaMKII, and CREB at 6 h and 7 d after microwave exposure. (**C**–**D**) The protein expression levels of GluN1, GluN2A, and GluN2B at 6 h and 7 d after microwave exposure. (**E**–**G**) Quantitative analysis of PSD-95, CaMKII, and CREB. (**H**–**J**) Quantitative analysis of GluN1, GluN2A, and GluN2B. Compared with the S group, * indicates *P* < 0.05, and ** indicates *P* < 0.01. Compared with the L5 group, ^#^ indicates *P* < 0.05, and.^##^ indicates *P* < 0.01

The results of western blotting for NMDAR subunits and downstream signalling pathway molecules at 6 h and 7 d after microwave exposure are shown in Fig. 5A–D, and the statistical analysis is shown in Fig. 5E–J. At 6 h after microwave exposure, compared with the S group, the levels of PSD-95 (*P* = 0.021, *n* = 3), CREB (*P* = 0.003, *n* = 3), GluN1 (*P* = 0.038, *n* = 4) and GluN2B (*P* = 0.008, *n* = 4) in the L5 group were reduced; the levels of GluN1 (*P* = 0.013, *n* = 4), GluN2A (*P* = 0.004, *n* = 4) and GluN2B (*P* = 0.008, *n* = 4) in the L30 group were reduced; and the levels of CREB (*P* = 0.026, *n* = 3), GluN1 (*P* = 0.002, *n* = 4), GluN2A (*P* = 0.001, *n* = 4) and GluN2B (*P* = 0.000, *n* = 4) in the L50 group were reduced. Compared with the L5 group, the levels of GluN2A (*P* = 0.019, *n* = 4) and GluN2B (*P* = 0.000, *n* = 4) in the L30 group were reduced; the levels of GluN2A (*P* = 0.004, *n* = 4) and GluN2B (*P* = 0.000, *n* = 4) in the L50 group were reduced; and the levels of PSD-95 (*P* = 0.001, *n* = 3), CaMKII (*P* = 0.044, *n* = 3) and CREB (*P* = 0.001, *n* = 3) in the L30 group were increased. Compared with the L30 group, the levels of PSD-95 (*P* = 0.015, *n* = 3) and CREB (*P* = 0.007, *n* = 3) in the L50 group were increased. At 7 d after microwave exposure, there were no significant changes in the abundance of NMDAR subunits or downstream signalling molecules (*P* > 0.05, *n* = 4).

## Discussion

Numerous studies have shown that electromagnetic radiation of a certain intensity can cause injuries to the brain, heart, reproductive organs, and other organs, among which the brain is an especially consequential target (Zhu et al. 2015; Hinrikus et al. 2017; Kesari et al. 2018). At present, there are few studies on injury caused by L-band microwave radiation on the nervous system, and the dose-dependent effect is not clear. Therefore, in this study, rats were exposed to 1.5-GHz microwaves with average power densities of 5 mW/cm^2^, 30 mW/cm^2^, and 50 mW/cm^2^ to explore the dose-dependent effect of 1.5-GHz microwave radiation on spatial memory impairment, structural damage and related molecular changes.

The most significant effect of electromagnetic radiation on the central nervous system was the impairment of learning and memory ability (Deshmukh et al. 2015; Shahin et al. 2015). The MWM is a common behavioural paradigm used to evaluate the learning and memory function of rats (Nunez 2008). The influence of microwave radiation on learning and memory function is still controversial. Most scholars found that microwaves could induce the impairment of learning and memory function, and other studies believed that electromagnetic radiation had a positive effect on learning and memory function (Koivisto et al. 2000; Sienkiewicz et al. 2000; Arendash et al. 2010; Mortazavi et al. 2013, Sharma and Shukla 2020). In a previous study by our laboratory, Wistar rats were exposed to S-band electromagnetic radiation with average power densities of 0, 5, 10, and 50 mW/cm^2^ at 2.856 GHz for 6 min. In the MWM navigation experiment, the AEL of rats in the 10 mW/cm^2^ and 50 mW/cm^2^ groups was significantly prolonged, and there were no significant changes in the AEL of rats in the 5 mW/cm^2^ group (Wang et al. 2013a). In this study, the MWM results showed that there were no significant changes in the spatial memory ability of rats in the L5 group. At 6 h, 1 d, and 2 d after radiation, the AEL of rats in the L50 group was prolonged, and spatial memory was impaired. Our previous paper also found that spatial memory impairment was most pronounced in the early stage after microwave exposure and that there was no obvious difference during the late stage after microwave exposure (Zhu et al. 2021). At 3 d after microwave exposure, there was no significant difference in AEL among the groups. The spatial probe test was performed at 4 d after microwave exposure to further evaluate the impairment of spatial memory. The results of the spatial probe test showed that the number of platform crossings and the time of platform quadrant in 1 min decreased and that spatial memory extraction was impaired in the L30 and L50 groups. The findings were interesting because microwaves might participate in the real-time or short-term regulation of brain function, which should be given enough attention. Therefore, the injury of L-band microwaves of 1.5 GHz on the spatial memory of rats was similar to that of S-band microwaves, and the injury was aggravated with the increase in dose.

EEG signals are an important neuro-electrophysiological index reflecting brain activity. The α and β waves normally occur when the brain is awake or excited. When the functional state of the brain is suppressed, slow waves with frequencies below 8 Hz occur, namely, δ waves and θ waves (Van Paesschen et al. 2007). Some researchers irradiated rats with microwaves at 30 mW/cm^2^. At 7 d–14 d after exposure, the θ wave and δ wave power increased, which was consistent with the results of this study(Zhao et al. 2020). In our experiment, EEG was measured at 6 h, 7 d, 14 d, and 28 d after exposure. The experimental results showed that there were no significant changes in EEG activity in the L5 group compared with the S group. The α wave power in the L30 group decreased at 6 h after exposure, and the θ wave and δ wave power increased at 6 h after exposure. The θ wave and δ wave power of the L50 group increased after microwave radiation, the increased amplitude was greater than that in the L30 group, and the duration was longer. In general, the EEG activity of rats in the L30 and L50 groups was inhibited. As the average power density increased, the inhibition of EEG activity increased as well. Thus, there was a negative dose-dependent effect between 1.5-GHz microwaves and EEG activity in rats.

After microwave exposure under certain conditions, the microscopic and ultramicroscopic structure of the hippocampus can change. This damage to the hippocampal tissue structure was mainly focused on the CA3 and DG regions (Zhi et al. 2017; Hao et al. 2018). Previous studies found that after microwave exposure, the nuclei of hippocampal neurons in rats showed pyknosis and deep staining, the eosinophilic cytoplasm was enhanced, and apoptosis increased(Karimi et al. 2018). Under an electron microscope, mitochondrial swelling, disordered ridge arrangement, thickening of the postsynaptic membrane density, and a reduction in the number of synaptic vesicles could be observed (Wang et al. 2015b). Quantitative analysis of H&E staining showed that 1.5-GHz microwaves could cause varying degrees of damage to hippocampal tissue structure. With increasing power, the injury became increasingly serious. At 6 h after microwave exposure, the tissue structure began to be injured, the injury was the most serious at 7 d after microwave exposure, and the damage began to recover at 14 d after microwave exposure. Moreover, combining the results regarding the effects of microwave exposure from our group (Zhu et al. 2021), we believe that the molecular parameters changed first, then the functional indexes changed, and the histological changes occurred last, which met the general rules of disease occurrence and development. Therefore, according to the histological results, we chose to observe the dose-dependent effect of 1.5-GHz microwaves on hippocampal ultrastructure at 7 d after microwave exposure. The ultrastructure of the hippocampus was observed under an electron microscope. The degree of injury was related to power. The PSD thickness was quantitatively analysed. When the power increased, the PSD thickness increased; this dose-dependent effect was readily observable.

NMDAR is an ionic glutamate receptor, and its subunit composition affects channel activity and downstream signals (Gladding and Raymond 2011, Zhou et al. 2015). PSD-95, CaMKII, and CREB are downstream molecules of NMDAR and important postsynaptic signal transduction proteins in the nervous system (Gardoni et al. 2006). They are of great significance in maintaining synaptic plasticity and regulating normal neural function. Blot K (Blot et al. 2015) used MK-801 to block NMDARs in the medial prefrontal cortex (mPFC), and rats showed impairment of mPFC-dependent cognitive flexibility and spatial memory. The use of glutamate receptor agonists ameliorated the spatial memory impairment induced by MK-801. Other studies have found that blocking NMDARs in nonhuman primates inhibited nerve cell firing and impaired working memory (Wang et al. 2013b). Wang (Wang et al. 2015a) irradiated rats with a frequency of 2.856 GHz and an average power density of 50 mW/cm^2^ for 6 min, and the expression levels of GluN1 and GluN2B decreased significantly. Other studies have found that the PSD-95 content in primary hippocampal neurons decreases after exposure to 1800 MHz microwave radiation(Xu et al. 2006). In this study, we established a dose-dependent model of the effect of microwave radiation, and the hippocampal levels of NMDAR-related proteins after microwave exposure were quantified by a fully automated western blotting system. The results showed that the concentrations of PSD-95 and CREB in the L5 group and CREB in the L50 group decreased at 6 h after microwave exposure. At 7 d after exposure, the injury was recovered, and the protein levels were not significantly different from their normal values. The concentrations of the NMDAR subunits GluN1, GluN2A, and GluN2B decreased at 6 h after 1.5-GHz microwave exposure. There was a dose-dependent effect; with increasing doses of radiation, the protein level decreased. At 7 d after exposure, the injury was recovered, and the protein levels were not significantly different from normal. A previous paper found that NMDARs protect against synaptic plasticity damage to primary hippocampal neurons by microwave radiation (Wang et al. 2018). It appears to us that the change in tissue structure had no direct relationship with NMDAR or downstream molecules. Our analysis of the tissue structure suggested that neurons had pyknotic and hyperchromatic changes, which might mediate the process of cell apoptosis after microwave exposure. The ultrastructure results showed that PSD thickness increased, and NMDARs were mainly expressed on the membrane, which was not directly related to the increase in postsynaptic density. Since there were many proteins in the postsynaptic region, which is a complex regulatory mechanism, it could not be proven that the changes in NMDAR consequences after microwave exposure were related to tissue structure changes. The specific mechanism still needs to be further studied.

In general, we found that, after 1.5-GHz microwave exposure, spatial memory was impaired, EEG activity was inhibited, the hippocampal structure was impaired, and the concentrations of NMDAR subunits and downstream signalling molecules were changed. Microwave impairment was reversible. At 6 h after exposure, spatial memory was impaired, EEG activity was inhibited, the molecular level of the NMDAR signalling pathway was changed, and the tissue structure was damaged. At 7 d after exposure, spatial memory and the NMDAR signalling pathway returned to normal, and the tissue structure was the most seriously damaged. Therefore, we believe that spatial memory impairment and EEG inhibition after 1.5-GHz microwave exposure may be caused by changes in the abundance of NMDAR subunits and downstream molecules in the NMDAR signalling pathway. After 1.5-GHz microwave exposure, the changes in molecular levels preceded the change in function, and the change in function preceded the change in structure. At 28 d after 1.5-GHz microwave exposure, the hippocampal structure returned to normal, which was consistent with the adult hippocampal regeneration cycle reported in a previous paper (Urbach and Witte 2019). In addition, there was a dose-dependent effect between 1.5-GHz microwaves and injury. The higher the dose, the more serious the injury and the longer the recovery time. Spatial memory ability, EEG activity, and hippocampal structure were altered, and the specific mechanism needs to be further discussed.

Our study revealed the role of NMDAR subunits and the NMDAR signalling pathway in the dose-dependent impairment of spatial memory by 1.5-GHz microwaves, which provides a basis for further research on the dose-dependent effect of microwave damage and forms of protection against such damage.

## Conclusion

Exposure to a 1.5-GHz microwave with an average power density of 5, 30, and 50 mW/cm^2^ for 6 min induces varying degrees of spatial memory impairment, hippocampal structure abnormalities, and changes in protein levels in rats (dose-dependent effect).

## Recommendation

This study explored the dose-dependent effect of 1.5 GHz microwave, found changes in cognitive function, histological structure and protein level, and laid a foundation for the subsequent dose-dependent effect of microwave. In the future, we suggest to do more work in the field of dose-dependent effect of microwave, further explore its specific mechanism, clarify the dynamic relationship between dose and effect, and extend it to the application of microwave protection.

## Supplementary Information

Below is the link to the electronic supplementary material.

Supplementary file1 (DOCX 34 KB)

## Data Availability

The data and materials used during the current study are available from the corresponding author on reasonable request.
