# Supplementary material for: The dose-dependent effect of 1.5-GHz microwave exposure on spatial memory and the NMDAR pathway in Wistar rats
Source: Environ Sci Pollut Res Int. 2022 Dec 27;30(13):37427–39. doi: 10.1007/s11356-022-24850-4 (PMC9792922; doi:10.1007/s11356-022-24850-4)
Supplement: Supplementary file 1 — Supplementary file1 (DOCX 34 KB) [file 11356_2022_24850_MOESM1_ESM.docx]

**Statistic data in Table1.**

| Table | Panel | Comparison | P-value | DF (df) | t |
| --- | --- | --- | --- | --- | --- |
| 1 | S | Rectal temperature before and after microwave exposure | 0.573 | 3 | 0.631 |
| 1 | S | Body surface temperature before and after microwave exposure | 0.851 | 3 | 0.204 |
| 1 | L5 | Rectal temperature before and after microwave exposure | 0.497 | 3 | 0.770 |
| 1 | L5 | Body surface temperature before and after microwave exposure | 0.893 | 3 | 0.146 |
| 1 | L30 | Rectal temperature before and after microwave exposure | 0.391 | 3 | 1.000 |
| 1 | L30 | Body surface temperature before and after microwave exposure | 0.630 | 3 | 0.535 |
| 1 | L50 | Rectal temperature before and after microwave exposure | 0.109 | 3 | 2.263 |
| 1 | L50 | Body surface temperature before and after microwave exposure | 0.705 | 3 | 0.417 |

**Statistic data for graphs in Figures.**

| Figure | Panel | Comparison | P-value | DF (df) | F |
| --- | --- | --- | --- | --- | --- |
| 1 | A | 6h: ANOVA | 0.009 | (3,39) | 4.466 |
|  |  | 6h: S vs. L5 | 0.552 |  |  |
|  |  | 6h: S vs. L30 | 0.079 |  |  |
|  |  | 6h: S vs. L50 | 0.002 |  |  |
|  |  | 6h: L5 vs. L30 | 0.99 |  |  |
|  |  | 6h: L5 vs. L50 | 0.008 |  |  |
|  |  | 6h: L30 vs. L50 | 0.123 |  |  |
|  |  | 1d: ANOVA | 0.039 | (3,39) | 3.090 |
|  |  | 1d: S vs. L5 | 0.312 |  |  |
|  |  | 1d: S vs. L30 | 0.254 |  |  |
|  |  | 1d: S vs. L50 | 0.005 |  |  |
|  |  | 1d: L5 vs. L30 | 0.894 |  |  |
|  |  | 1d: L5 vs. L50 | 0.057 |  |  |
|  |  | 1d: L30 vs. L50 | 0.076 |  |  |
|  |  | 2d: ANOVA | 0.009 | (3,39) | 4.481 |
|  |  | 2d: S vs. L5 | 0.055 |  |  |
|  |  | 2d: S vs. L30 | 0.386 |  |  |
|  |  | 2d: S vs. L50 | 0.001 |  |  |
|  |  | 2d: L5 vs. L30 | 0.276 |  |  |
|  |  | 2d: L5 vs. L50 | 0.146 |  |  |
|  |  | 2d: L30 vs. L50 | 0.014 |  |  |
|  |  | 3d: ANOVA | 0.137 | (3,39) | 1.966 |
|  |  | 7d: ANOVA | 0.617 | (3,39) | 0.603 |
|  |  | 14d: ANOVA | 0.613 | (3,39) | 0.609 |
|  |  | Tests of Between-Subjects Effects of Overall Group | <0.001 | (3,36) | 11.669 |
|  |  | S vs. L5 | 0.014 |  |  |
|  |  | S vs. L30 | 0.012 |  |  |
|  |  | S vs. L50 | <0.001 |  |  |
|  |  | L5 vs. L30 | 0.943 |  |  |
|  |  | L5 vs. L50 | 0.002 |  |  |
|  |  | L30 vs. L50 | 0.003 |  |  |
| 1 | B | 6h: ANOVA | 0.407 | (3,39) | 0.993 |
|  |  | 1d: ANOVA | 0.066 | (3,39) | 2,609 |
|  |  | 2d: ANOVA | 0.138 | (3,39) | 1.953 |
|  |  | 3d: ANOVA | 0.476 | (3,39) | 0.849 |
|  |  | 7d: ANOVA | 0.140 | (3,39) | 1.946 |
|  |  | 14d: ANOVA | 0.721 | (3,39) | 0.447 |
|  |  | Tests of Between-Subjects Effects of Overall Group | 0.394 | (3,36) | 1.022 |
| 1 | C | ANOVA | 0.002 | (3,39) | 5.819 |
|  |  | S vs. L5 | 0.581 |  |  |
|  |  | S vs. L30 | 0.003 |  |  |
|  |  | S vs. L50 | 0.003 |  |  |
|  |  | L5 vs. L30 | 0.012 |  |  |
|  |  | L5 vs. L50 | 0.012 |  |  |
|  |  | L30 vs. L50 | 1.000 |  |  |
| 1 | D | ANOVA | 0.010 | (3,39) | 4.360 |
|  |  | S vs. L5 | 0.169 |  |  |
|  |  | S vs. L30 | 0.002 |  |  |
|  |  | S vs. L50 | 0.009 |  |  |
|  |  | L5 vs. L30 | 0.068 |  |  |
|  |  | L5 vs. L50 | 0.177 |  |  |
|  |  | L30 vs. L50 | 0.618 |  |  |
| 2 | A | 6h: ANOVA | 0.117 | (3,19) | 2.297 |
|  |  | 6h: S vs. L5 | 0.095 |  |  |
|  |  | 6h: S vs. L30 | 0.041 |  |  |
|  |  | 6h: S vs. L50 | 0.036 |  |  |
|  |  | 6h: L5 vs. L30 | 0.660 |  |  |
|  |  | 6h: L5 vs. L50 | 0.620 |  |  |
|  |  | 6h: L30 vs. L50 | 0.955 |  |  |
|  |  | 7d: ANOVA | 0.367 | (3,19) | 1.129 |
|  |  | 14d: ANOVA | 0.215 | (3,19) | 1.664 |
|  |  | 28d: ANOVA | 0.777 | (3,19) | 0.368 |
|  |  | Tests of Between-Subjects Effects of Overall Group | 0.646 | (3,16) | 0.565 |
| 2 | B | 6h: ANOVA | 0.725 | (3,19) | 0.444 |
|  |  | 7d: ANOVA | 0.746 | (3,19) | 0.413 |
|  |  | 14d: ANOVA | 0.886 | (3,19) | 0.213 |
|  |  | 28d: ANOVA | 0.843 | (3,19) | 0.274 |
|  |  | Tests of Between-Subjects Effects of Overall Group |  | (3,16) | 0.449 |
| 2 | C | 6h: ANOVA | 0.008 | (3,19) | 5.625 |
|  |  | 6h: S vs. L5 | 0.110 |  |  |
|  |  | 6h: S vs. L30 | 0.008 |  |  |
|  |  | 6h: S vs. L50 | 0.001 |  |  |
|  |  | 6h: L5 vs. L30 | 0.203 |  |  |
|  |  | 6h: L5 vs. L50 | 0.047 |  |  |
|  |  | 6h: L30 vs. L50 | 0.424 |  |  |
|  |  | 7d: ANOVA | 0.015 | (3,19) | 4,718 |
|  |  | 7d: S vs. L5 | 0.341 |  |  |
|  |  | 7d: S vs. L30 | 0.056 |  |  |
|  |  | 7d: S vs. L50 | 0.002 |  |  |
|  |  | 7d: L5 vs. L30 | 0.295 |  |  |
|  |  | 7d: L5 vs. L50 | 0.019 |  |  |
|  |  | 7d: L30 vs. L50 | 0.149 |  |  |
|  |  | 14d: ANOVA | 0.724 | (3,19) | 0.445 |
|  |  | 28d: ANOVA | 0.572 | (3,19) | 0.688 |
|  |  | Tests of Between-Subjects Effects of Overall Group | 0.007 | (3,16) | 5.734 |
|  |  | S vs. L5 | 0.152 |  |  |
|  |  | S vs. L30 | 0.015 |  |  |
|  |  | S vs. L50 | 0.001 |  |  |
|  |  | L5 vs. L30 | 0.245 |  |  |
|  |  | L5 vs. L50 | 0.026 |  |  |
|  |  | L30 vs. L50 | 0.228 |  |  |
| 2 | D | 6h: ANOVA | 0.004 | (3,19) | 6.809 |
|  |  | 6h: S vs. L5 | 0.182 |  |  |
|  |  | 6h: S vs. L30 | 0.004 |  |  |
|  |  | 6h: S vs. L50 | 0.001 |  |  |
|  |  | 6h: L5 vs. L30 | 0.101 |  |  |
|  |  | 6h: L5 vs. L50 | 0.023 |  |  |
|  |  | 6h: L30 vs. L50 | 0.446 |  |  |
|  |  | 7d: ANOVA | 0.082 | (3,19) | 2.683 |
|  |  | 7d: S vs. L5 | 0.405 |  |  |
|  |  | 7d: S vs. L30 | 0.100 |  |  |
|  |  | 7d: S vs. L50 | 0.016 |  |  |
|  |  | 7d: L5 vs. L30 | 0.386 |  |  |
|  |  | 7d: L5 vs. L50 | 0.085 |  |  |
|  |  | 7d: L30 vs. L50 | 0.359 |  |  |
|  |  | 14d: ANOVA | 0.759 | (3,19) | 0.396 |
|  |  | 28d: ANOVA | 0.558 | (3,19) | 0.714 |
|  |  | Tests of Between-Subjects Effects of Overall Group | 0.003 | (3,16) | 7.171 |
|  |  | S vs. L5 | 0.474 |  |  |
|  |  | S vs. L30 | 0.005 |  |  |
|  |  | S vs. L50 | 0.001 |  |  |
|  |  | L5 vs. L30 | 0.024 |  |  |
|  |  | L5 vs. L50 | 0.006 |  |  |
|  |  | L30 vs. L50 | 0.496 |  |  |
| 3 | H | ANOVA | <0.001 | (3,19) | 35..504 |
|  |  | S vs. L5 | 0.017 |  |  |
|  |  | S vs. L30 | <0.001 |  |  |
|  |  | S vs. L50 | <0.001 |  |  |
|  |  | L5 vs. L30 | <0.001 |  |  |
|  |  | L5 vs. L50 | <0.001 |  |  |
|  |  | L30 vs. L50 | 0.111 |  |  |
| 4 | I | ANOVA | <0.001 | (3,19) | 61.196 |
|  |  | S vs. L5 | <0.001 |  |  |
|  |  | S vs. L30 | <0.001 |  |  |
|  |  | S vs. L50 | <0.001 |  |  |
|  |  | L5 vs. L30 | <0.001 |  |  |
|  |  | L5 vs. L50 | <0.001 |  |  |
|  |  | L30 vs. L50 | 0.611 |  |  |
| 5 | E | 6h: ANOVA | 0.008 | (3,11) | 8.220 |
|  |  | 6h: S vs. L5 | 0.021 |  |  |
|  |  | 6h: S vs. L30 | 0.083 |  |  |
|  |  | 6h: S vs. L50 | 0.305 |  |  |
|  |  | 6h: L5 vs. L30 | 0.001 |  |  |
|  |  | 6h: L5 vs. L50 | 0.115 |  |  |
|  |  | 6h: L30 vs. L50 | 0.015 |  |  |
|  |  | 7d: ANOVA | 0.745 | (3,15) | 0.416 |
| 5 | F | 6h: ANOVA | 0.168 | (3,11) | 2.179 |
|  |  | 6h: S vs. L5 | 0.590 |  |  |
|  |  | 6h: S vs. L30 | 0.106 |  |  |
|  |  | 6h: S vs. L50 | 0.969 |  |  |
|  |  | 6h: L5 vs. L30 | 0.044 |  |  |
|  |  | 6h: L5 vs. L50 | 0.616 |  |  |
|  |  | 6h: L30 vs. L50 | 0.099 |  |  |
|  |  | 7d: ANOVA | 0.689 | (3,15) | 0.500 |
| 5 | G | 6h: ANOVA | 0.003 | (3,11) | 10.996 |
|  |  | 6h: S vs. L5 | 0.003 |  |  |
|  |  | 6h: S vs. L30 | 0.394 |  |  |
|  |  | 6h: S vs. L50 | 0.026 |  |  |
|  |  | 6h: L5 vs. L30 | 0.001 |  |  |
|  |  | 6h: L5 vs. L50 | 0.192 |  |  |
|  |  | 6h: L30 vs. L50 | 0.007 |  |  |
|  |  | 7d: ANOVA | 0.246 | (3,15) | 1.577 |
| 5 | H | 6h: ANOVA | 0.012 | (3,15) | 5.583 |
|  |  | 6h: S vs. L5 | 0.038 |  |  |
|  |  | 6h: S vs. L30 | 0.013 |  |  |
|  |  | 6h: S vs. L50 | 0.002 |  |  |
|  |  | 6h: L5 vs. L30 | 0.584 |  |  |
|  |  | 6h: L5 vs. L50 | 0.131 |  |  |
|  |  | 6h: L30 vs. L50 | 0.310 |  |  |
|  |  | 7d: ANOVA | 0.906 | (3,15) | 0.183 |
| 5 | I | 6h: ANOVA | 0.002 | (3,15) | 8.823 |
|  |  | 6h: S vs. L5 | 0.407 |  |  |
|  |  | 6h: S vs. L30 | 0.004 |  |  |
|  |  | 6h: S vs. L50 | 0.001 |  |  |
|  |  | 6h: L5 vs. L30 | 0.019 |  |  |
|  |  | 6h: L5 vs. L50 | 0.004 |  |  |
|  |  | 6h: L30 vs. L50 | 0.438 |  |  |
|  |  | 7d: ANOVA | 0.199 | (3,15) | 1.810 |
| 5 | J | 6h: ANOVA | <0.001 | (3,15) | 36.385 |
|  |  | 6h: S vs. L5 | 0.008 |  |  |
|  |  | 6h: S vs. L30 | <0.001 |  |  |
|  |  | 6h: S vs. L50 | <0.001 |  |  |
|  |  | 6h: L5 vs. L30 | <0.001 |  |  |
|  |  | 6h: L5 vs. L50 | <0.001 |  |  |
|  |  | 6h: L30 vs. L50 | 0.855 |  |  |
|  |  | 7d: ANOVA | 0.972 | (3,15) | 0.075 |
